# 10 year risk of death in adolescents with learning disability or autism in England: a national cohort study using linked health and education data from ECHILD

**DOI:** 10.23889/ijpds.v9i5.2555

**Published:** 2024-09-18

**Authors:** Ania Zylbersztejn, Isobel Ward, Katie Harron, Ruth Gilbert

**Affiliations:** 1 UCL Great Ormond Street Institute of Child Health; 2 NIHR Great Ormond Street Hospital Biomedical Research Centre; 2 Office for National Statistics

## Background

Transition from adolescent to adult health and education services is a vulnerable period for young people with learning disability (LD) or autism spectrum disorders (ASD), yet there is a lack of information on their risk of death in that period.

## Methods

We developed an English national cohort of 11-year-olds enrolled in state schools between 2009/10-20014/15, followed-up until their 22nd birthday, death or 31st August 2020 using linked health, mortality and education data from the ECHILD database. LD and ASD were indicated using diagnoses from hospital admissions or if LD or ASD were a reason for special educational needs provision in education records before age 11. We estimated crude mortality rates and compared with unaffected peers using rate ratios (RR).

## Results

The cohort comprised 3,080,516 young people (52% boys), of whom 4,589 (0.15%) died (crude mortality rate: 0.17/1000 person-years, 95% CI: 0.16-0.17/1000).

45,174 (1.5%) young people were autistic (85% boys; 15% with co-existing LD), of whom 113 died (mortality rate: 0.29/1000 person-years, 95%CI: 0.24-0.35/1000). Mortality was 1.7 times higher for autistic young people compared to their peers (RR: 1.7, 95% CI: 1.4-2.1). Among 40,264 (1.3% of all) young people with LD (63% boys; 17% with co-existing ASD) there were 812 deaths (mortality rate: 2.31/1000, 95%CI: 2.15-2.47/1000PY). They had 17 times higher risk of death (RR: 16.4, 95%CI: 15.2-17.7).

## Conclusions

Young people with LD/ASD are more likely to die than their peers. Findings on disparities within these groups according to co-existing comorbidities, sex and socioeconomic factors will be reported.

